# High-throughput single-cell profiling of B cell responses following inactivated influenza vaccination in young and older adults

**DOI:** 10.18632/aging.204778

**Published:** 2023-06-26

**Authors:** Meng Wang, Ruoyi Jiang, Subhasis Mohanty, Hailong Meng, Albert C. Shaw, Steven H. Kleinstein

**Affiliations:** 1 Program in Computational Biology and Bioinformatics, Yale University, New Haven, CT 06510, USA; 2 Department of Immunobiology, Yale School of Medicine, New Haven, CT 06510, USA; 3 Section of Infectious Diseases, Department of Internal Medicine, Yale School of Medicine, New Haven, CT 06510, USA; 4 Department of Pathology, Yale School of Medicine, New Haven, CT 06510, USA

**Keywords:** B cell receptor, repertoire, clonal expansion, aging, single-cell RNA-seq

## Abstract

Seasonal influenza contributes to a substantial disease burden, resulting in approximately 10 million hospital visits and 50 thousand deaths in a typical year in the United States. 70 - 85% of the mortality occurs in people over the age of 65. Influenza vaccination is the best protection against the virus, but it is less effective for the elderly, which may be in part due to differences in the quantity or type of B cells induced by vaccination. To investigate this possibility, we sorted pre- and post-vaccination peripheral blood B cells from three young and three older adults with strong antibody responses to the inactivated influenza vaccine and employed single-cell technology to simultaneously profile the gene expression and the B cell receptor (BCR) of the B cells. Prior to vaccination, we observed a higher somatic hypermutation frequency and a higher abundance of activated B cells in older adults than in young adults. Following vaccination, young adults mounted a more clonal response than older adults. The expanded clones included a mix of plasmablasts, activated B cells, and resting memory B cells in both age groups, with a decreased proportion of plasmablasts in older adults. Differential abundance analysis identified additional vaccine-responsive cells that were not part of expanded clones, especially in older adults. We observed broadly consistent gene expression changes in vaccine-responsive plasmablasts and greater heterogeneity among activated B cells between age groups. These quantitative and qualitative differences in the B cells provide insights into age-related changes in influenza vaccination response.

## INTRODUCTION

Older adults are at increased risk for morbidity and mortality from infectious diseases and show impaired responses to vaccination. Influenza and COVID-19 are two examples of infections in which 70 - 85% of mortality occurs in adults over age 65 [1, 2]. Protection against influenza virus infection from vaccination is markedly diminished in older adults, with approximately a 50% decrease in protection compared to young individuals [3]. Age-related alterations in innate and adaptive immune function, or immunosenescence, are critical to such poor vaccine responses. Aging of the innate immune system is notable for a chronic inflammatory state that is associated with impaired functions of innate immune pattern recognition receptors such as the retinoic acid-induced gene-I (RIG-I) and Toll-like receptors (TLRs) [4]. The adaptive immune system in older adults is notable for intrinsic signaling alterations [5] and impaired T cell production as a result of thymic involution, such that the T cell pool is maintained almost exclusively by peripheral expansion in older adults [6]. Studies of the human B cell compartment have revealed an age-associated decrease in expression of activation-induced cytidine deaminase (AID) linked to diminished expression of the E47 transcription factor [7].

A burst of plasmablasts is often observed in peripheral blood 7 days after influenza vaccination. Previous B cell repertoire sequencing studies have also shown clonal expansion of plasmablasts in both young and older adults 7 days after influenza vaccination, with older adults having fewer lineages and increased oligoclonality in BCR repertoires [8–11]. In addition, activated B cells, a subset of antigen-specific cells that are committed to the memory B cell lineage, are observed to expand and peak around 2 weeks post-vaccination in young adults [12, 13]. However, since studies have focused analyses on plasmablasts or bulk B cell populations in young adults, how activated B cells in older adults respond to influenza vaccination is not well studied.

Apart from differences in clonal expansion in response to influenza vaccination, previous repertoire studies also revealed a decrease in somatic hypermutation (SHM) level for plasmablasts in older adults, which may be associated with decreased adaptability of antibody response [8, 9]. However, these analyses examined only plasmablasts; and the response to influenza vaccination in adults is by definition a memory response since nearly all adults have had primary immune responses to the influenza virus via infection or vaccination previously. An analysis of SHM frequency for different B cell compartments and isotypes in response to influenza vaccination remains lacking.

Previous studies also identified transcriptional signatures of influenza vaccination response. Bulk sequencing studies have found plasmablast gene signatures in peripheral blood 7 days following influenza vaccination for both young and older adults [7, 14, 15]. A recent single-cell sequencing study found that the expanded clones 7 days post-vaccination exhibit an activated memory B cell gene expression profile in a young adult [16]. These studies indicate an overall frequency increase of plasmablasts and activated memory B cells following vaccination, but it is unclear whether there are changes within these B cell compartments following vaccination and whether the changes are different between young and older adults.

While older adults generally have more extensive prior exposure to influenza than young individuals and might be expected to have a more broad-based B cell memory of the influenza virus, the basis for the decreased plasmablast response in older, compared to young adults [17] remains incompletely understood. To gain insight into functional age-related differences in the B cell lineage response to vaccination, we have carried out paired single-cell RNAseq and BCR sequencing in a group of young and older adults, where both groups showed evidence for a protective antibody response following seasonal influenza vaccination.

## RESULTS

### Single-cell profiling of the influenza vaccination response

**Figure 1:**
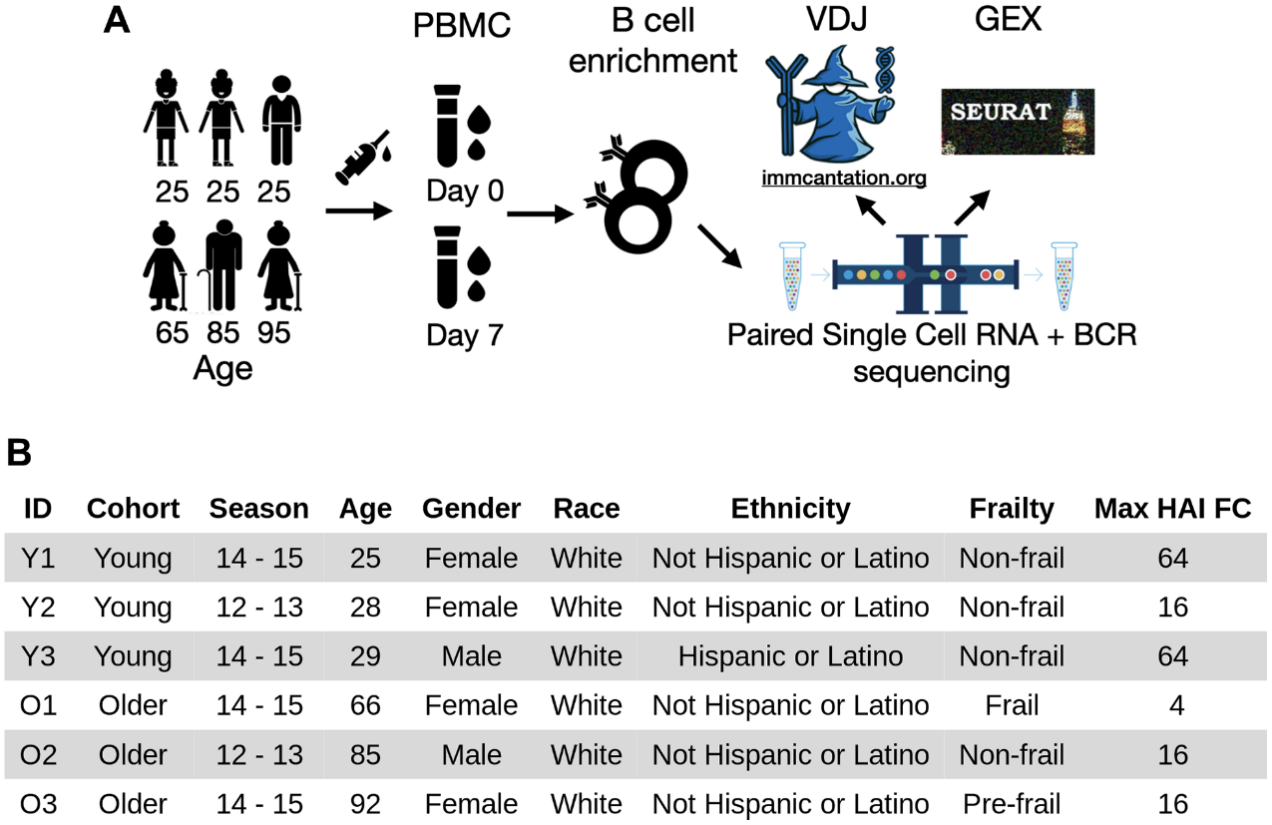
**Experimental workflow and subject demographics.** (**A**) PBMC samples were collected from three young and three older adults (midpoint of the age range shown) before and seven days after vaccination. The samples were negatively enriched for B cells. Paired single-cell RNA and B-cell receptor sequencing were then performed. The resulting heavy and light V(D)J sequences were analyzed by immcantation, and gene expression data were analyzed using Seurat. (**B**) Demographic information of the six subjects. The subjects were selected based on at least 4-fold increase in hemagglutination inhibition titers of at least one vaccine strain at day seven.

To understand the age-group difference in B cells underlying a successful influenza vaccination response, we profiled the B cells enriched from PBMC samples prior to receiving vaccination (day 0, D0) and 7 days post-vaccination (D7) from three young and three older subjects using 10x Genomics paired single-cell RNAseq and BCR sequencing (Figure 1A). All six subjects were selected from a previously described cohort recruited at Yale University [14] and each responded to the standard-dose seasonal influenza vaccine with at least a 4-fold increase of post-vaccine HAI titer for at least one vaccine strain at day 28 (Figure 1B). Specifically, three of the six subjects (Y1, Y3, O3) had at least 4-fold responses to all strains in the vaccine, while the other three (Y2, O1, O2) had at least 4-fold responses to only one strain in the vaccine (Supplementary Figure 1). There was no significant difference in HAI titers before vaccination between age groups for each vaccine strain (two-sided Wilcoxon rank-sum test, p > 0.05). The older adults had a lower level of antibody response (max HAI FC ranges between 4 and 16) compared with the young adults (max HAI FC ranges between 16 and 64).

**Figure 2:**
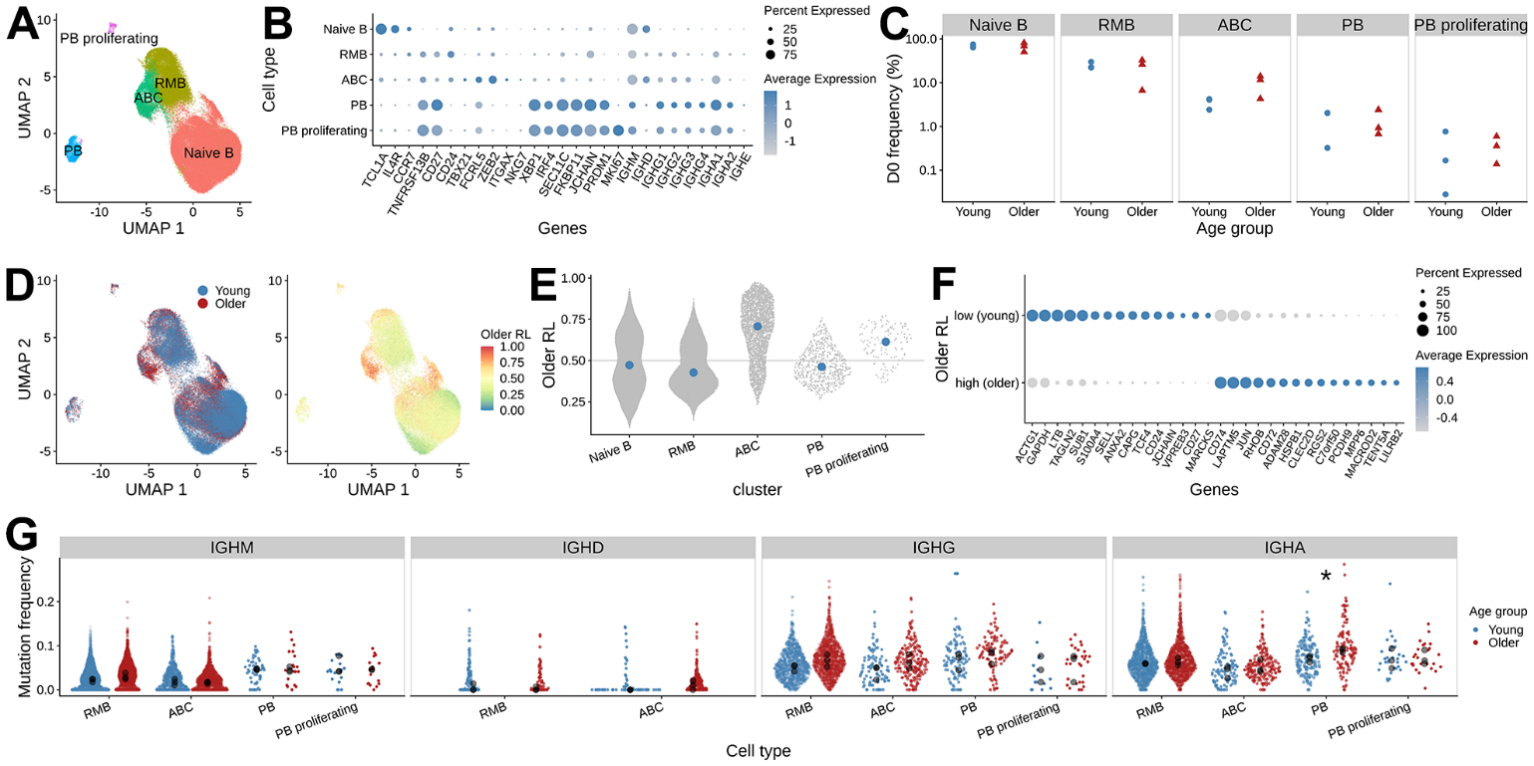
**Differences in B cell gene expression between young and older adults prior to vaccination.** (**A**) UMAP visualization of single-cell RNAseq data, colored by B cell subset assignment (RMB: resting memory B cells, ABC: activated B cells, PB: plasmablast). (**B**) Expression of the marker genes used for B cell subset assignment. Dot plot showing average log-normalized expression of B cell subset marker genes and the fraction of cells expressing the genes in each B cell cluster. (**C**) The frequency of B cell subsets at pre-vaccination. The x-axis, along with shape and color of the dots, indicates the age group, and the y-axis represents the frequency of B cell subsets in each sample. (**D**) Pre-vaccination (D0) differential abundance analysis of B cells between young and older adults. The left panel is colored by the age group label. The right panel is colored by the MELD score that indicates older adults-associated relative likelihood; the higher the value, the more likely that the cells with the given gene expression profile are from the older subject samples. (**E**) MELD score distribution for the B cell subsets. (**F**) Differentially expressed genes between ABCs with high and low MELD score, were selected from gene set enrichment analysis using enrichR. (**G**) Mutation frequency distribution of the heavy chain V segments. The mean SHM frequency of each subject is indicated as black dots for each isotype and B cell subset, colored by age group. Wilcoxon rank-sum test was performed to test for the difference in mean mutation frequency between age groups (*: adjusted p-values < 0.05).

We applied reference-based cell type annotation on gene expression data from 117,278 single cells using Azimuth and identified 100,745 B cells (85.9% of all cells). Further clustering on the B cell data found five B cell clusters: naïve B cells, resting memory B cells (RMB), activated B cells (ABC), plasmablasts (PB), and proliferating plasmablasts (PB proliferating) (Figure 2A, 2B). We found that 89.5 % of these B cells were associated with BCR heavy chains captured in the V(D)J data, and we focused all subsequent analyses on the 90,133 B cells with both gene expression and BCR information. Overall, we analyzed 62,197 naïve B cells, 19,319 resting memory B cells, 6,336 activated B cells, 1,944 plasmablasts, and 337 proliferating plasmablasts.

### Higher frequency of activated B cell subset in older adults at pre-vaccination

To evaluate pre-vaccination differences between age groups, we first compared the relative abundance of the D0 B cell subsets. We did not detect significant differences in the abundance of any of the five B cell subsets between the age groups (Figure 2C, two-sided Wilcoxon rank-sum test, p > 0.05). However, it was noteworthy that the frequency of ABCs was higher in 2 of the 3 older subjects (11.60 % and 13.98 %) compared with 3 young subjects (ranging from 2.41 % - 4.21 %) (Supplementary Figure 2).

This cluster-level analysis lacks the resolution to identify the differentially abundant subpopulations that may exist within each of these B cell types. We utilized the MELD (manifold enhancement of latent dimensions [18]) algorithm for each cell type and computed a relative likelihood score that quantifies how likely each cell is to be observed in older relative to young adults given its gene expression (Figure 2D). The higher the MELD relative likelihood score a cell has, the more likely that cells with similar gene expression patterns come from older adults. This analysis identified cells within the ABCs that had an extremely high relative likelihood score (Figure 2E), indicating that they are present mostly in older subjects. We focused our subsequent analysis on these differentially abundant cells within the ABCs.

To characterize the ABC subpopulations that were enriched in older adults, we applied MELD with Vertex Frequency Clustering (VFC) to find three clusters of ABCs. The choice of the number of VFC clusters is based on the uniformity of the transcriptomic profile and the enrichment of age group labels within the clusters (Supplementary Figure 3). We took the VFC cluster with the highest average relative likelihood score (older adults enriched) and the lowest average relative likelihood score (young adults enriched) and filtered for cells from the corresponding age groups to perform differential gene expression analysis. In total, we compared 587 young adults’ ABCs from the VFC score low cluster (ABC subset enriched in young adults) and 536 older adults’ ABCs from the VFC score high cluster (ABC subset enriched in older adults). We used the pseudo-bulk approach to aggregate the cell counts within each sample and applied limma to find differentially expressed genes. We found 547 upregulated genes with an FDR-adjusted p-value < 0.05 in the ABCs from older adults. We annotated these genes with enrichR [19] and found genes enriched in pathways including AP-1 transcription factor networks (FOS, JUN, ATF3) and antigen processing and presentation (HLA genes). We also found 433 genes associated with ABCs that have higher expression in young adults. Enrichment analysis of these genes identified associations with cytoplasmic ribosomal proteins, death receptor signaling (FAS, CASP8, CASP10), and caspase-mediated cleavage of cytoskeleton proteins (GSN, ADD1, VIM) (Figure 2F and Supplementary Figure 4). In summary, these analyses show that, prior to vaccination, ABCs in older adults exhibit a different gene expression profile from those in young adults.

### Higher pre-vaccination mutational load in older adults

Previous BCR repertoire analysis reported higher SHM levels of IgG B cells prior to vaccination in older adults [11]. With the paired gene expression data, we were able to examine the pre-vaccination SHM frequencies between age groups separately for each isotype and cell type. We computed heavy chain mutation frequency using IGHV gene sequences from BCR sequencing data and tested for differences in the mean IGHV SHM frequency between age groups for each cell type and isotype (Figure 2G). We found older subjects tended to have higher mutation frequency compared with young subjects across several cell types and isotypes. In particular, IgA+ PB showed significantly higher mean SHM frequency in older subjects (two-sided Wilcoxon rank-sum test, p = 0.04). In summary, young and older adults differ in the mutation load of B cell repertoire at baseline for some subsets.

### Young adults mount a more clonal response compared with older adults

**Figure 3:**
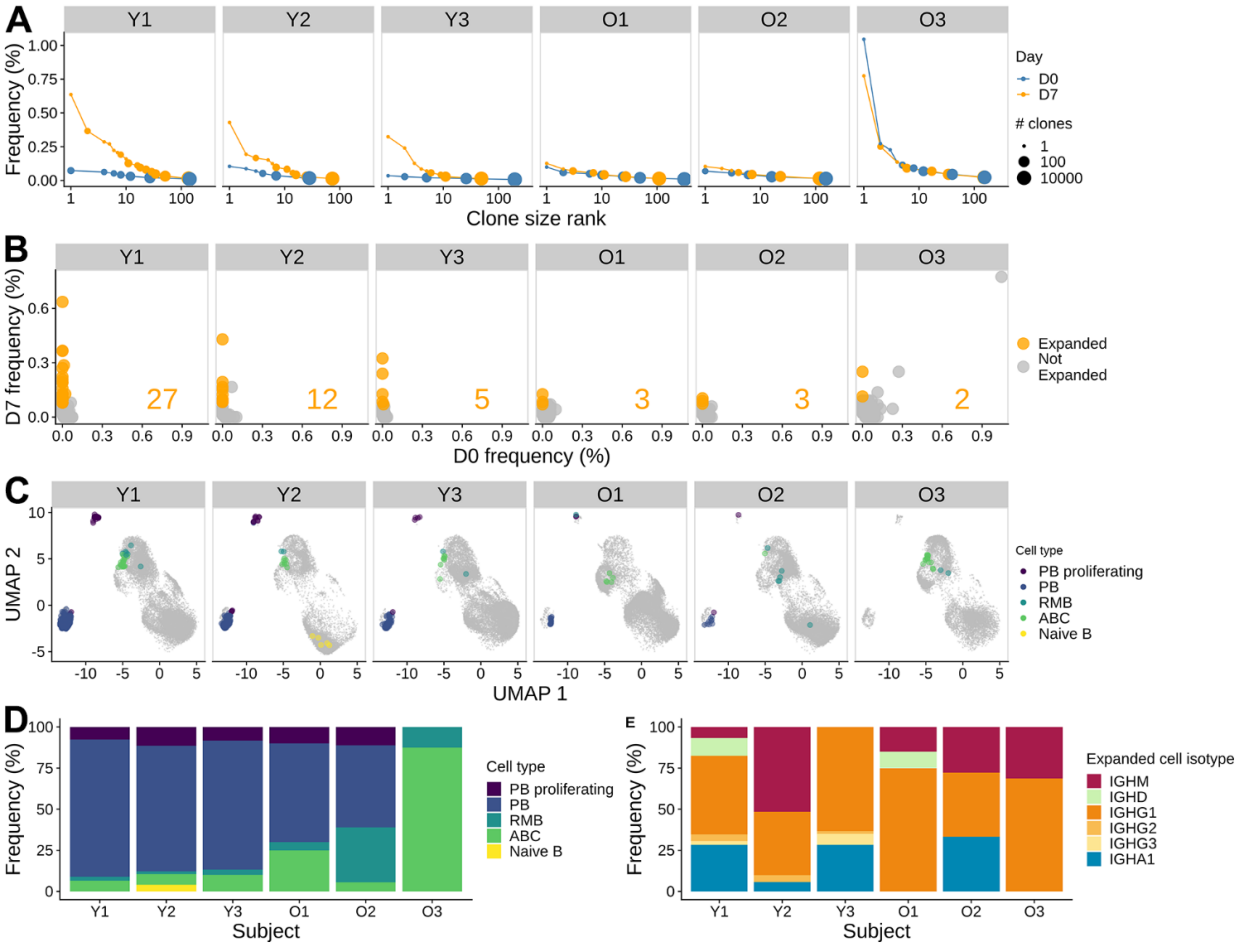
**Influenza vaccine induces more clonal expansion in young adults, and the induced clones are more skewed towards memory in older adults.** (**A**) B cell clone size distribution. The size of the dots indicates the number of clones. The x-axis indicates the rank of the clone size, with the rank of 1 being the largest. The y-axis indicates the relative abundance of the clone within the sample. The color indicates the time points. (**B**) Significantly expanded clones at day 7 post-vaccination. The frequencies of individual clones pre-vaccination and at day 7 post-vaccination were visualized by scatter plot. Fisher's exact test was used to identify significantly expanded clones, highlighted in orange. The number of significantly expanded clones was annotated in the plot for each subject. (**C**) Significantly expanded clones visualized on a UMAP, highlighted with color indicating the cell type. (**D**) The cell subset composition of the expanded clones at day 7. (**E**) Isotype composition of the expanded clones for day 7 samples.

Influenza vaccination has been associated with the induction of clonally-expanded plasmablasts that generally peak around seven days post-vaccination [20]. Previous studies have found significantly reduced quantities of vaccine-specific plasmablasts circulating one week after vaccination in older subjects [17]. To compare the age-group difference in B cell response, we identified clones based on the similarity of their heavy chain nucleotide sequence and light chain gene usage. We found that pre-vaccination repertoires had very few large clones in both young and older individuals. The largest clone for Y1, Y2, Y3, O1, O2, and O3 pre-vaccination comprised 0.07, 0.10, 0.04, 0.10, 0.07, and 1.05 % of their repertoire, respectively. In contrast, we found many large clonal expansions in young adults at D7 (Figure 3A), with the largest clone consisting of 0.64, 0.43, and 0.32 % of the repertoire for Y1 - Y3, respectively. In contrast, older adults showed little difference in clone size distribution at D7 compared to D0, with the largest clone comprising 0.13, 0.10, and 0.78 % of the repertoire for O1 - O3, respectively.

To identify individual B cell clones that were expanded following influenza vaccination, we performed Fisher's exact test to determine whether a given clone significantly increased its abundance at D7 compared to D0. We identified 2 - 27 expanded clones in each individual (Figure 3B). The largest expanded clone comprised 0.64 % of the repertoire at D7 in Y1. As expected from the cell type abundance analysis, more expanded clones were identified in the young subjects compared with the older subjects (5 - 27 clones in young vs. 2 - 3 clones in older subjects). The single largest clone observed at D7 in subject O3 was also related to the largest clone observed at D0 and was not significantly expanded. In the other subjects, expanded clones were present at very low frequency or not detected at D0. Overall, we found that vaccination elicited expanded clones in all 6 subjects; however, the numbers of expanded clones identified were higher in young subjects than in older subjects.

### Expanded clones are a mix of plasmablasts, activated B cells, and resting memory B cells, with a bias towards plasmablasts in young and activated B cells in older subjects

To characterize the expanded clones, we examined the cell types and isotypes of the clone members. We found that the expanded clones were a mix of plasmablasts, ABCs, and RMBs (Figure 3C). In young subjects, expanded clones were dominated by plasmablasts (78.3 - 83.5 % of cells in expanded clones at D7). The composition of expanded clones in the older subjects was more diverse, with increased proportions of ABCs and RMBs; one of the subjects (O3) did not have any plasmablasts among cells in expanded clones (Figure 3D). We also examined the isotype of expanded clones. We observed that the BCRs in cells from expanded clones were predominantly IgG1 for all subjects (38.5 - 68.8 %), except for Y2, who had a high proportion of IgM+ cells (51.6 %) (Figure 3E). All of the young subjects and one of the older subjects also had IgA1+ cells as part of the expanded clones (5.74 - 33.3 %). In summary, expanded clones in all subjects were a mix of plasmablasts, activated B cells, and resting memory B cells, with a large bias towards IgG1+ plasmablasts in young adults.

**Figure 4:**
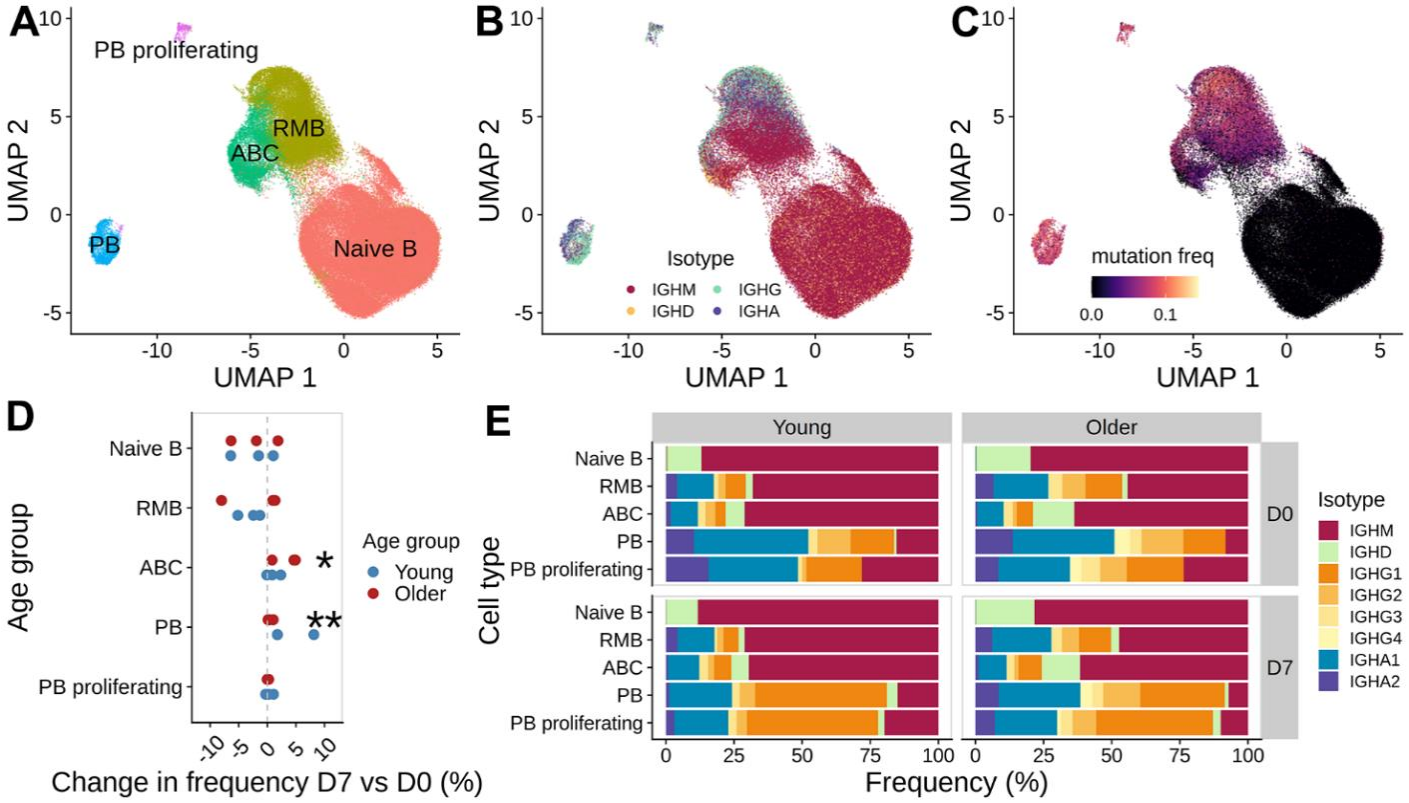
**Analysis of B cell subpopulations before and after vaccination.** UMAP of gene expression data colored with (**A**) B cell subset labels, (**B**) isotype information from the V(D)J sequences, and (**C**) mutation frequency in the V segment of heavy chain BCRs. (**D**) Difference in the frequency of B cell subset between day 7 and day 0 for each subject, colored by age group (*: p < 0.1, **: p < 0.05). (**E**) Isotype composition for each cell subset, separated by age group and time point.

To corroborate the observations from expanded clone analysis, we also examined the overall changes in cell type, isotype, and mutation frequency between vaccination time points (Figure 4A–4C). Consistent with the observations from expanded clone analysis, we observed a significant increase in plasmablast abundance at D7 (Wilcoxon rank-sum test, p = 0.031), with young adults having a larger increase than older adults (Figure 4D and Supplementary Figure 2). We also saw a moderate, albeit not significant, increase in the frequency of ABCs at D7 (Wilcoxon rank-sum test, p = 0.063), with a higher increase in older adults than young subjects (Figure 4D and Supplementary Figure 2). The cell frequency changes were accompanied by shifts in isotype composition. We detected significant changes in isotype composition between D7 and D0 for PB cells in all young subjects and one older adult O2 (Chi-square test for homogeneity, FDR-adjusted p-values < 0.05). The change in isotype composition involves an increase in IgG1 frequency in the PB compartments at D7 (Figure 4E); the difference in % IgG1+ PB between D7 and D0 ranged from 20.68 - 45.38 % in young adults and 9.26 - 24.75 % in older adults. Finally, we examined the SHM frequency difference between cells at D7 and D0 for each isotype and cell type. We computed the log-fold change in the mean SHM frequency of IGHV genes (Supplementary Figure 5) and found no significant change in SHM frequency comparing young and older subjects (two-sided Wilcoxon rank-sum test, p > 0.05). In summary, we observed an increase in plasmablast abundance post-vaccination in both age groups, with young adults having a larger response; older adults also had an increase in ABC abundance at day 7 but there was no significant difference in mutation load observed.

### Consistent change in gene expression was observed for vaccine-responsive plasmablasts but not activated B cells between age groups

**Figure 5:**
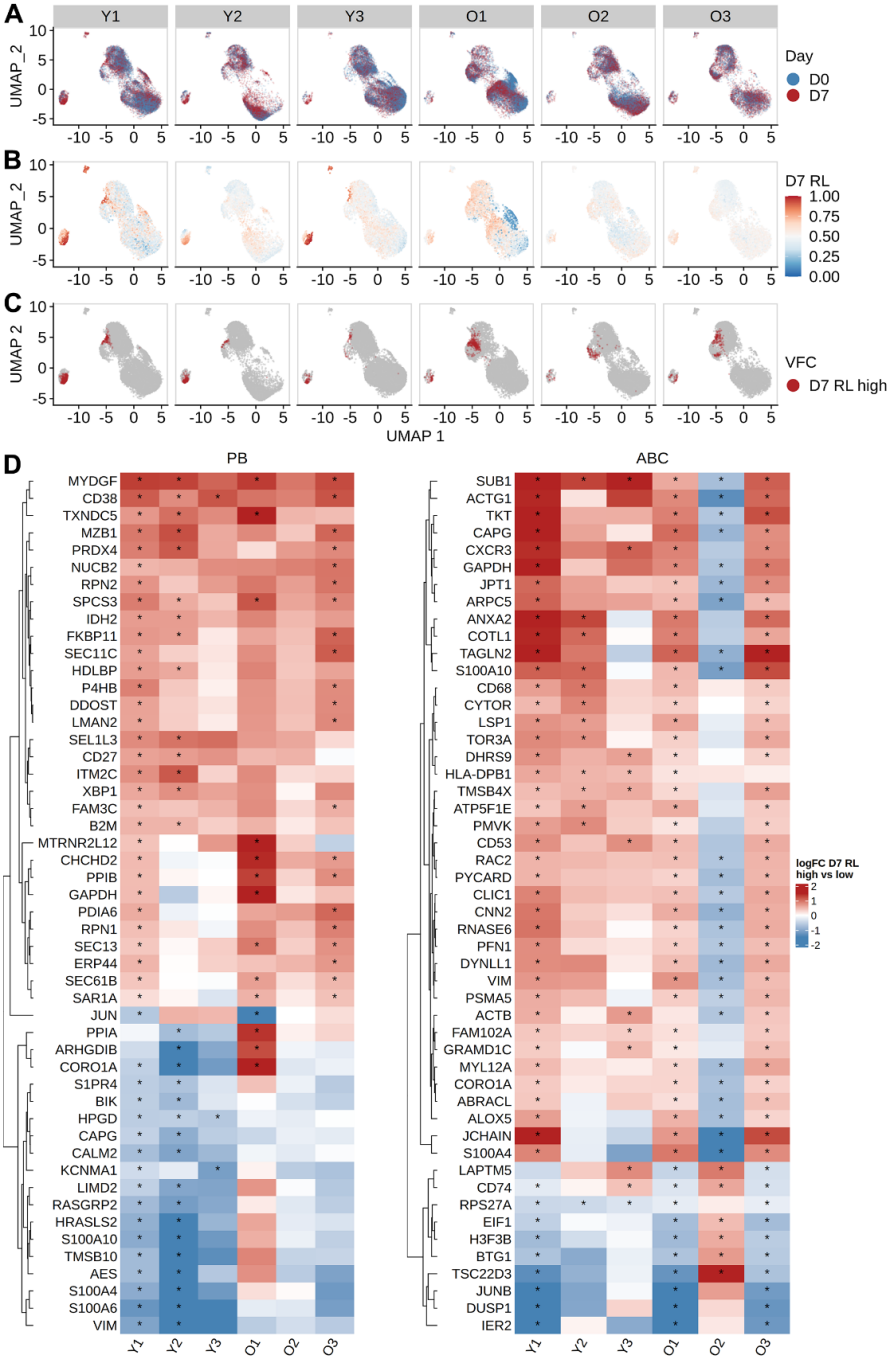
**Identifying vaccine-responsive subpopulations within plasmablasts and activated B cells.** MELD differential abundance analysis to find cell subpopulations changing abundance between day 0 and day 7. UMAP of gene expression data colored by (**A**) the day label of the cells, (**B**) signal filtered by MELD, and (**C**) the vertex frequency clustering to find subclusters of PB and ABC with differential abundance between day 0 and day 7. (**D**) Top 50 differentially expressed genes based on average absolute log FC between day 0 and day 7 were shown for each subject. Wilcoxon rank-sum test was used to select differentially expressed genes by comparing high MELD score (day 7-like) and low MELD score (day 0-like) clusters for each subject (FDR-adjusted p-value < 0.05). Genes that significantly differ between high and low MELD score clusters, and have an average absolute log-2 fold change greater than 0.3 in at least one subject, were selected for heatmap visualization. The color in the heat map indicates the log-2 fold change of count values between high and low MELD score clusters. Asterisks indicate an FDR-adjusted p-value smaller than 0.05.

The frequency of cells in expanded clone analysis and changes in cell type frequency gave inconsistent estimates of vaccine-responsive B cells: for example, expanded clones were < 1 % of D7 ABCs in all older participants, but the frequency of ABCs increased by 16.2 - 28.6 % at D7 from D0 in these individuals (Supplementary Figure 2A). We reasoned that the expanded clone analysis was likely to underestimate the total vaccination response since it excludes small clones recruited into the response. To more precisely define the B cell subpopulations that were “vaccine-responsive”, we applied MELD and vertex frequency clustering on each B cell subset. We chose to cluster the data into 3 clusters based on the homogeneity of MELD score and day labels within the clusters at given cluster resolution (Supplementary Figure 8–10). We first computed the MELD relative likelihood score of D7 against D0 from the day label of the cells (Figure 5A); a higher score indicates an increased likelihood to observe a given cell at D7 than at D0. We found subpopulations with high D7 relative likelihood scores located within plasmablasts for both age groups and within ABCs for older subjects (Figure 5B). This indicates the presence of transcriptionally similar subpopulations enriched post-vaccination within both the PB and ABC cell type clusters. We further used vertex frequency clustering to identify subpopulations of ABCs and PBs that had high values of D7-associated relative likelihood (Figure 5C) and defined them as “vaccine-responsive”. The frequency of vaccine-responsive ABC cells was higher in older (14.05 - 17.23 %) than young (7.04 - 16.58 %) adults at D7, whereas the frequency of vaccine-responsive PB cells was higher in young (38.02 - 53.27 %) than older (16.41 - 39.86 %) adults at D7 (Supplementary Table 3). The estimate of vaccine-responsive cells was usually higher than the frequency of expanded clones for each cell subset (Supplementary Figure 6).

Next, we evaluated vaccine-induced gene signatures in PB, ABC, and RMB by performing differential gene expression analysis. Within each cell type, we compared the vaccine-responsive cells (i.e., those in the cluster with the highest D7-associated relative likelihood at D7) with a control set of non-responding cells (selected as those in the cluster with the highest D0-associated relative likelihood at D0). It has been previously reported that comparing VFC clusters based on MELD recovers gene signatures more accurately than directly comparing two samples [18]. For PBs, we identified 402 genes with a significant change in expression value between vaccine-responsive vs non-responding cells in either young or older subjects (FDR-adjusted p-values from Wilcoxon rank-sum tests summarized across either young or older subjects using Fisher’s method < 0.05, and median average log2 fold change > 0.25), with expression signatures that were highly similar between young and older adults (Figure 5D left). The median average log FC change of expression values of the two age groups were correlated (Pearson correlation coefficient = 0.58), with young adults showing a larger change than the older adults (Supplementary Figure 7). To determine the biological relevance of these genes, we performed gene set enrichment analysis using enrichR. We saw an up-regulation of multiple genes related to translation (SEC61 genes, EIF genes, ribosomal protein genes) and proteasome degradation (PSMD genes). The expression of genes encoding chaperone proteins such as MZB1 and HSP90B1 was also increased; notably, these proteins have been reported to be critical effectors for the Blimp1 transcription factor implicated in the regulation of plasma cell function [21]. Additional upregulated genes include those implicated in chemokine signaling (CXCR3, CCR2, CD27) and protein export (SPCS3, SEC11C) pathways. Upregulation of CAV1, a component of lipid rafts, was also observed. For ABCs, we identified 375 genes with a significant change in expression value between vaccine-responsive and non-responding ABCs in the young or older populations (Figure 5D right). The pattern of response was relatively consistent across individuals but showed more variability than the PB response, particularly for subject O2. Notably, gene set enrichment analysis showed an enrichment of genes related to interferon signaling (IRF5, IRF7, IFI30, IFITM1, HLA genes) and antigen-activated BCR generation of second messengers (BTK, BLK, PLCG2). The median log2 fold change of gene expression is also lower in the older subjects than in young subjects (Supplementary Figure 7). For RMBs, we identified 363 genes with a significant change in expression between vaccine-responsive and non-responsive RMB cells in the young or older populations. There were no consistent patterns of change in expression between young and older individuals (Supplementary Figure 11). There was no correlation in the median log2 fold change after vaccination in these genes between young and older adults (Supplementary Figure 7). Gene set enrichment analysis showed that the differentially expressed genes were enriched in cytoplasmic ribosomal proteins and actin cytoskeleton regulation (ACTB, ACTG1, ARPC genes, ITG genes) (Supplementary Figure 11). To summarize, we observed a vaccine-induced gene signature in PB and ABCs. This signature was similar between age groups, with higher variability between subjects in ABCs.

### The oldest subject has a large persistent resting memory B cell clone

**Figure 6:**
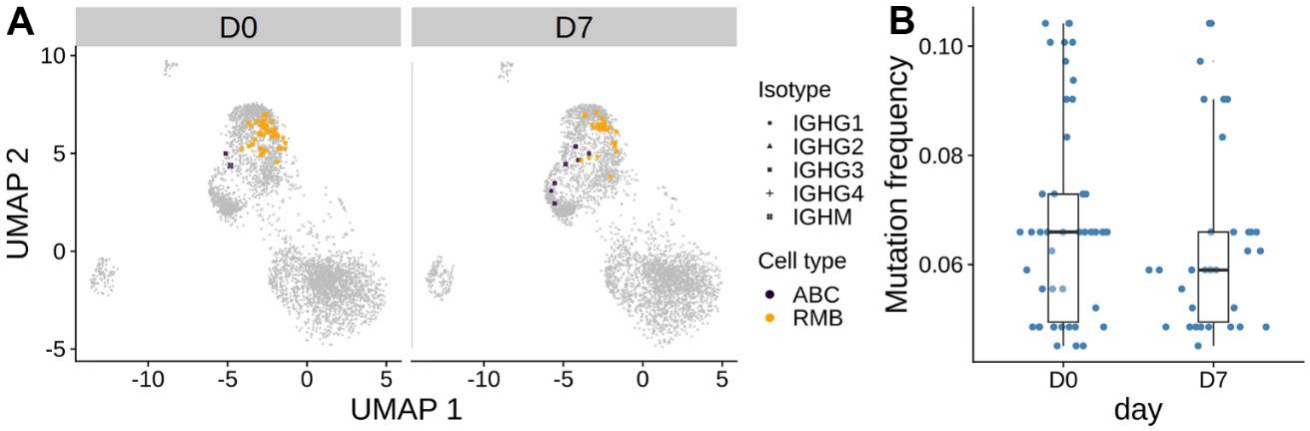
**The largest persistent clone in the oldest subject O3.** (**A**) The cells from the large persistent clone from the oldest subject are highlighted on the gene expression UMAP plot. The color indicates the cell type. The clone consists of only IgG cells. (**B**) Mutation frequency in the heavy chain V-segment of the cells within the clone between days was visualized in the box plot. No significant difference between days was observed by the Wilcoxon rank-sum test.

In the oldest subject O3, we observed a large clone present at both D0 and D7. This clone was much larger compared with the largest clones in any of the other subjects at any time point (frequency of 1.05 % and 0.774 % at D0 and D7, respectively) (Figure 3A). Further analysis revealed that the heavy chain of this clone used IGHV1-69 and IGHJ4. The clone was mostly composed of resting memory B cells of isotype IgG (Figure 6A). The clone did not significantly expand after vaccination and there was no significant change in SHM frequency between D0 and D7 (Figure 6B, two-sided Wilcoxon rank-sum test p > 0.05, date randomization test [22] p > 0.05), which indicates that the clone is unlikely to be part of the vaccination response. We performed a differential gene expression of cells in this clone with other IgG+ RMB in O3 at D0. The cells in the clone had significantly higher expression of cytoplasmic ribosomal genes and lower expression of MHC class II antigen presentation molecules than the other IgG D0 RMB (Supplementary Figure 12).

## DISCUSSION

Though decreased in incidence during the COVID-19 pandemic, influenza infection remains an important cause of morbidity and mortality in older adults, who account for as much as 70 - 85 % of influenza-associated mortality in the US [1]. Re-emergence of influenza infection following the COVID-19 pandemic is virtually certain, and the seasonal vaccine remains the mainstay of prevention efforts despite its substantially diminished effectiveness, particularly in older adults. To elucidate the effects of age on the B cell response to influenza vaccination, we carried out paired single-cell RNA-seq and BCR sequencing on enriched B cell samples prior to and 7 days following vaccination with standard-dose, seasonal inactivated influenza vaccine in young (age 20-30) and older (age 60-100) adults, all of whom showed at least a four-fold increase between pre- and post-vaccination HAI titer to at least one influenza strain in the vaccine. The use of standard-dose in both age groups allows for direct comparison of B cell response. We found evidence for an age-associated decrease in B cell vaccine response, with decreased B cell clonal expansion at D7 post-vaccination, in older individuals (Figure 3); such clonal expansion was dominated by plasmablasts in young individuals, compared to a more heterogeneous mix of plasmablasts, activated B cells and resting memory B cells in older adults. As a result, the expansion of plasmablasts post-vaccination (Figure 4D) was greater in young, compared to older adults, consistent with prior findings in studies of the age-associated decrease in B cell antibody response post-vaccination [17].

The use of single cell technology allows us to examine the SHM frequency in detail for each cell type and isotype. We observed higher pre-vaccine SHM frequency in the older across cell types in IgG and IgA compartment, compared to young adults (Figure 2G), which may reflect a more extensive prior infection and vaccination history in older adults. We also saw no significant change in SHM frequency in young and older adults for most B cell subsets at D7 post-vaccination; while an age-associated decrease in activation-induced cytidine deaminase (AID) expression has been found in B cells from older, compared to young individuals, it seems likely that germinal center responses are not fully developed seven days post-vaccination, making it difficult to detect a potential decrease in SHM (Figure 4F) [23].

The age-associated decrease in plasmablast expansion we observed in older, compared to young adults raises the question of whether functional alterations in plasmablasts could account for this difference. We analyzed gene expression signatures in plasmablasts and found that gene expression patterns were generally preserved in young and older adults (Figure 5); in contrast, greater heterogeneity in gene expression between young and older adults was observed in the ABC. In this context, influenza vaccination elicits the expansion of plasmablasts, as well as activated B cells which contribute to the memory B cell pool [12]. Taken together, our findings suggest that the age-related decrease in response following influenza vaccination could reflect functional alterations in activated B cells, since once generated, plasmablasts from young and older adults appear highly comparable at the gene expression level.

We observed a large, persistent clone in the oldest subject (O3) that consisted largely of resting memory B cells and did not expand from day 0 to day 7 post-vaccine (Figure 6). Such B cell clonal expansions have been previously described and correlate with age and EBV serologic status [24]. While we did not detect expansion of any PB clone in this participant, it is notable that this individual showed an at least 4-fold increase in post-vaccine HAI titer for all viral strains in the seasonal vaccine (Supplementary Figure 1). Because post-vaccine HAI titer was measured at day 28 post-vaccine, it seems likely that the plasmablast response in this individual was delayed past day 7.

A limitation of the current study is that it includes only a small number of subjects spanning a large age range, which leads to high variance and low statistical significance of many comparisons. Despite this limitation, we nevertheless were able to identify important commonalities and differences in the influenza vaccination response between young and older individuals. A potential limitation of this study is the use of an increase in the frequency of a clone or cells with transcriptional similarity as an indicator for response to the vaccine, which does not necessarily imply specificity to influenza virus. Typically, the massive burst of plasmablasts 7 days after influenza vaccination in peripheral blood is highly enriched for vaccine-specific cells. A previous study [12] reported that around 82 % of IgG+ antibody-secreting cells and 45 % of IgG+ activated B cells are specific against influenza virus among isotype-switched, actively proliferating B cells in peripheral blood 7 days after vaccination for healthy, young adults. However, bystander expansion of non-specific memory B cells has also been commonly observed [16, 20].

To summarize, we showed a quantitative difference in B cell response following vaccination between age groups, with expansion dominated by plasmablasts in the young, and activated B cells in older adults. The gene expression changes between young and older adults are highly similar in plasmablasts, indicating that the differences in the PB response between young and older adults are mostly in the quantity instead of the quality of B cells. This suggests that vaccines that can simply engage more cells would induce better short-term protection in older populations. On the other hand, improving the durability of protection may require activating different pathways, particularly in the ABC population. ABCs are likely the precursors of long-lived memory B cells and displayed heterogeneity in their gene expression between young and older adults. Overall, this study provides insights into the B cell vaccine response differences between young and older adults and may be beneficial to design more effective vaccines in the older age groups.

## MATERIALS AND METHODS

### Subject selection

We selected three young (20 to 30 years old) and three older (60 to 100 years old) influenza vaccine responders from a cohort recruited at Yale University as described previously [14]. The six subjects were from two influenza vaccination seasons: 2012 - 2013 (Y2, O2) and 2014 -2015 (Y1, Y3, O1, O3), and responded to the vaccine with at least a 4-fold increase in HAI titer at day 28 post-vaccination for at least one vaccine strain (Supplementary Figure 1). The 2012 - 2013 season vaccine strains included A/California/7/2009, A/Victoria/361/2011, and B/Wisconsin/1/2010. The 2014 - 2015 season vaccine was identical to that of the 2013-2014 season and included A/California/7/2009, A/Texas/50/2012, B/Brisbane/60/2008, and B/Massachusetts/2/2012.

### Emulsion-based single-cell library preparation

We isolated B cells from PBMCs using the EasySepTM Human Pan-B cell Enrichment Kit (immunomagnetic negative selection kit, StemCell) per the manufacturer’s instructions. We prepared single-cell emulsions and libraries using the Chromium Single-cell 5′ Reagent Kit for version 1 chemistry per the manufacturer’s protocol using the Chromium Controller (10x Genomics). The NovaSeq 6000 with 100x100 or 150x150 paired-end reads were used to sequence gene expression and BCR libraries respectively in the same manner as previously [25]. Base calls were converted to FASTQ sequences and demultiplexed using the Cell Ranger v3.1.0 mkfastq function. Demultiplexed FASTQ reads were aligned to the coding sequences of the GCRhg38 coding genome supplied by 10X Genomics. The sequence depth information for BCR and GEX data is available in Supplementary Tables 1, 2 respectively.

### Processing of 10x Genomics single-cell BCR reads

We removed sequences with nonproductive arrangements and filtered for cells with exactly one heavy chain sequence. B cell clones were inferred based on heavy chain sequences using hierarchical clustering with single-linkage [26] for each subject. Specifically, sequences were partitioned based on common V and J gene annotations and junction lengths. Within each partition, sequences whose junction sequences were within 0.09 normalized Hamming distances between each other were clustered as heavy chain clones. The cutoff was chosen based on a visual inspection of the bimodal distance-to-nearest distribution of hamming distances. The heavy chain clones were further divided based on light chain gene usage as the final clones. SHM frequency was computed using the “calcObservedMutations” function from SHazaM v1.0.2 package [27], which counts the number of nucleotide mismatches from the germline sequences of the heavy chain variable segments leading up to the CDR3.

### Processing of 10x Genomics single-cell 5′ gene expression data

To generate count matrices, barcode assignments, and feature calls, we used the Cell Ranger count subcommand. Seurat v4 [28] was used for gene expression analysis. We removed cells with fewer than 400 transcripts or with mitochondrial content of more than 15 % of all transcripts. We library-normalized and log-transformed the UMI counts and selected the top 2000 variable genes using the “FindVariableFeatures” function with the “vst” option. We removed immunoglobulin and T cell receptor-related genes from the list of highly variable genes so that their properties could be analyzed independently of cell type annotation. We centered and scaled the data, ran principal component analysis and took the first 50 principal components to generate the UMAP embeddings.

We first identified and removed non-B cells by annotating the cell type of each single cell using azimuth v0.4.5 with human PBMC reference v1.0.0 (celltype.l2 level annotation). Cells that were not annotated to one of the B cell subsets (B naive, B intermediate, B memory, plasmablast) were removed. We then clustered the B cell data using the “FindClusters” function with a resolution of 2 and manually annotated the cluster identities using a set of previously described marker genes for four different types of B cells based on the following set of markers [13]: XBP1, IRF4, SEC11C, FKBP11, JCHAIN, and PRDM1 for plasmablasts, with MKI67 used to identify a subset of proliferating plasmablasts; TCL1A, IL4R, CCR7, IGHM, and IGHD for naive B cells; TBX21, FCRL5, ITGAX, NKG7, ZEB2 for activated B cells; and TNFRSF13B, CD27 and CD24 for resting memory B cells. We merged the gene expression data with the BCR data by cell barcodes and filtered for cells with both data modalities.

### Differential abundance of pre-vaccination B cells between age groups

We applied MELD v1.0.0 [18] to the pre-vaccination B cells to identify B cell subpopulations that were different between young and older subjects at pre-vaccination. We built a cell-state graph from the first 100 PCA dimensions of the gene expression data with 50 nearest neighbors using the python graphtools package (https://github.com/KrishnaswamyLab/graphtools). We ran MELD on the cell-state graph using young/older labels as input and applied the MELD vertex frequency clustering (VFC) method to the ABC cluster to identify ABC subpopulations that were differentially abundant between age groups. The number of clusters was chosen by inspecting the similarity of gene expression and the uniformity of MELD relative likelihood score within the clusters. To identify gene signatures of age group differences, we performed pseudo-bulk differential expression between VFC clusters with the highest and lowest older adults-associated average likelihood score by aggregating the cell counts within each sample and applying limma v3.42.2 to find differentially expressed genes (Bonferroni corrected p-value < 0.05). Gene set enrichment analysis of differentially expressed genes was performed using EnrichR v2.1 [19].

### Identify significantly expanded clones

We computed the frequency of each clone at pre-vaccination and 7 days post-vaccination. To identify significantly expanded clones on day 7, we performed a one-sided Fisher’s exact test to test if the frequency of a given clone increases on day 7 using the “fisher.test” function in R with the alternative option set as “greater”. We corrected for multiple testing using the “p.adjust” function with the “BH” method and identified significantly expanded clones using a cutoff at 0.05.

### Identify vaccine-responsive cells

We applied MELD to gene expression to identify differentially abundant populations between pre-vaccination and 7 days post-vaccination for plasma B cell, activated B cell, and naive B cell clusters, separately for each subject. For each run, we computed the cell state graph using the first 100 PCA dimensions with KNN = 20. MELD was run on each cell state graph using the day labels to compute the sample-associated relative likelihood. To identify cell subpopulations similar in gene expression as well as similar response to vaccine at day 7, vertex frequency clustering was performed on the cell state graph and the sample-associated relative likelihood, for each of the clusters per subject. We chose three VFC clusters based on the similarity in gene expression and uniformity of the relative likelihood score of clusters by visualization. Differentially gene expression calculation was performed between the VFC clusters with the highest and the lowest day 7 associated relative likelihood using Seurat “FindAllMarkers” function. Gene set enrichment analysis of differentially expressed genes was performed using EnrichR.

### Evaluate change in somatic hypermutation frequency for the largest clone in O3

We used the R function “wilcox.test” to perform a two-sided Wilcoxon rank-sum test to test for the change in SHM frequency in the largest clone in O3 between day 0 and day 7. In addition, we constructed a lineage tree for the clone based on the BCR heavy chain sequences and performed a root-to-tip regression permutation test (correlationTest) to test whether there was a significant change in SHM frequency between timepoints using dowser v1.0.0 [22].

### Data availability

Single-cell RNA sequencing and V(D)J data have been deposited in NCBI’s Gene Expression Omnibus and are available at the GEO Series accession number GSE175524. Scripts to reproduce these analyses are available at: https://bitbucket.org/kleinstein/projects/src/master/Wang2023_Aging.

## Supplementary Material

Supplementary Figures

Supplementary Tables
