# Supplementary material for: High-throughput single-cell profiling of B cell responses following inactivated influenza vaccination in young and older adults
Source: Aging (Albany NY). 2023 Jun 26;15(18):9250–74. doi: 10.18632/aging.204778 (PMC10564424; doi:10.18632/aging.204778)
Supplement: Supplementary Figures [file aging-15-204778-s001.pdf]

SUPPLEMENTARY FIGURES

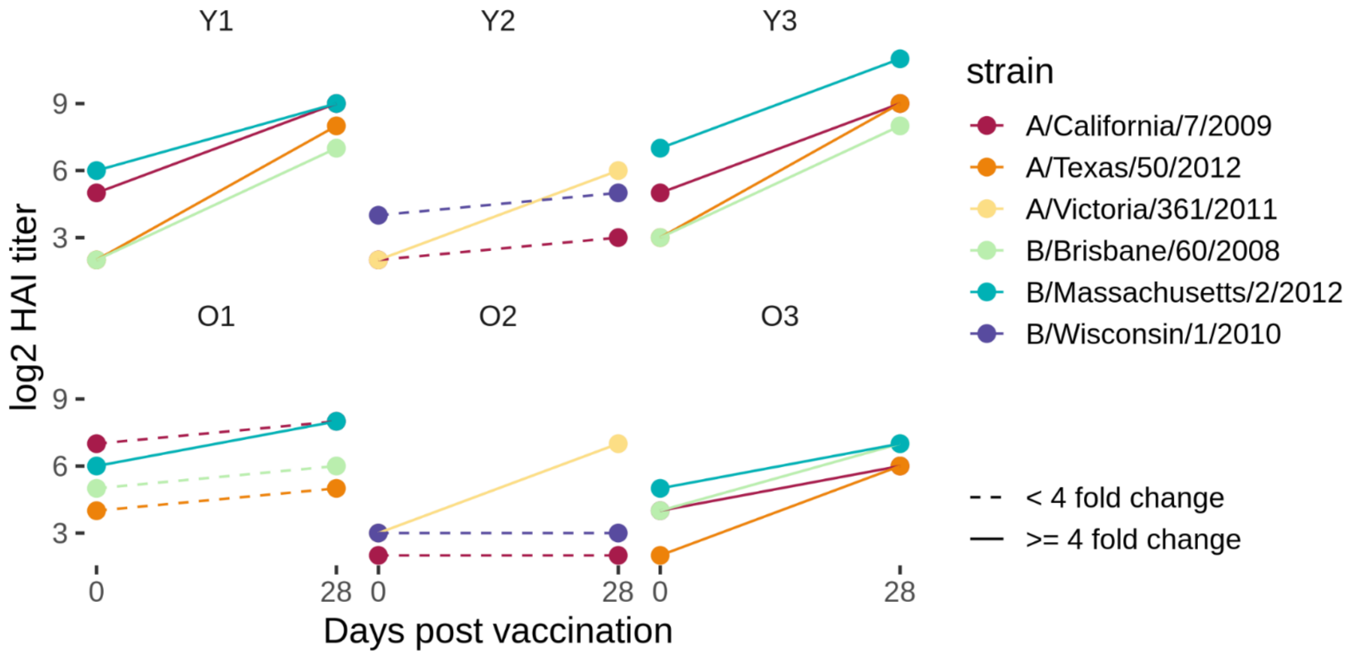

Supplementary Figure 1. Hemagglutination inhibition assay titers of vaccine strains pre-vaccination and day 7 post-vaccination. The x-axis indicates the post-vaccination time point. The y-axis indicates the log2 fold change in HAI titer. The color indicates the vaccine strain. Note that Y2 and O2 received the trivalent standard-dose influenza vaccine during the 2012-2013 season, while Y1, Y3, O1 and O3 received the 2014-2015 quadrivalent standard-dose vaccine.

A

Cluster frequency (%)	day	Y1	Y2	Y3	O1	O2	O3
Naive B	D0	63.81	70.33	75.12	80.72	68.55	50.41
Naive B	D7	57.13	71.19	73.56	74.42	66.63	52.24
RMB	D0	29.92	22.67	22.11	6.65	26.10	32.61
RMB	D7	25.17	20.44	20.86	7.95	27.05	24.61
ABC	D0	4.07	4.21	2.41	11.60	4.28	13.98
ABC	D7	6.46	4.13	3.24	16.24	5.12	18.91
PB	D0	2.04	2.02	0.32	0.67	0.93	2.41
PB	D7	10.02	3.78	2.06	1.05	1.05	3.42
PB proliferating	D0	0.17	0.77	0.03	0.36	0.14	0.59
PB proliferating	D7	1.22	0.47	0.28	0.34	0.15	0.82

B

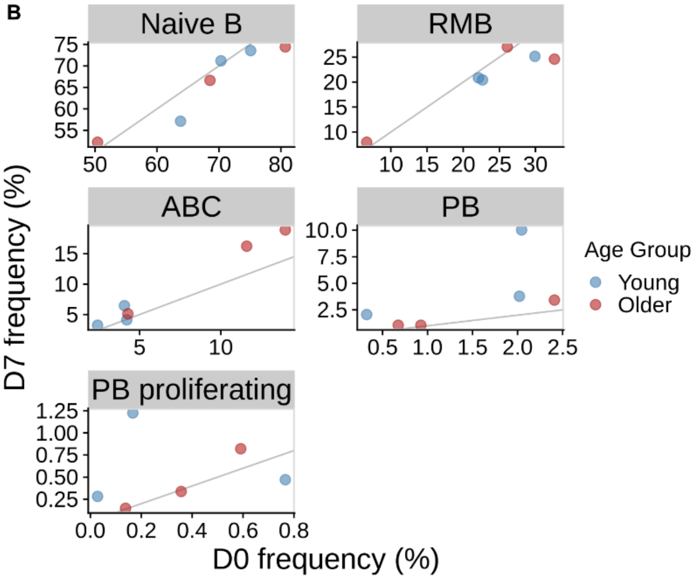

Supplementary Figure 2. B cell cluster frequency for each subject at each time point. (**A**) B cell cluster frequencies of each sample. (**B**) Scatter plot of the frequency of the cluster between D0 and D7. The gray diagonal line has a slope of 1 and an intercept of 0. Data points above the diagonal line are samples with increased frequency of the given cluster at D7.

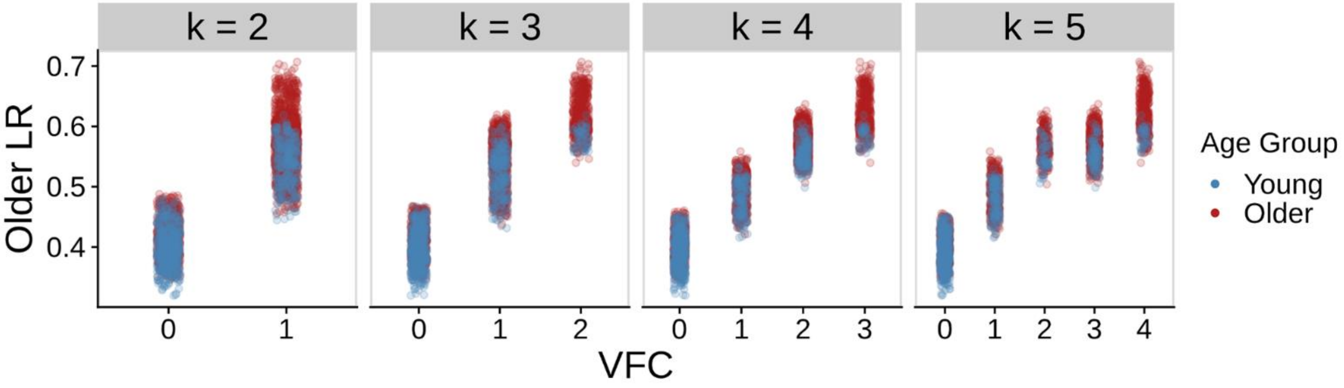

Supplementary Figure 3. Choice of the number of VFC clusters *k* to identify differentially abundant ABC subpopulations at pre-vaccination. The x-axis is the cluster id and the y-axis is the relative likelihood of observing the cell in older relative to young adults. The color indicates the age group labels. *k = 3* is the final choice of the number of clusters.

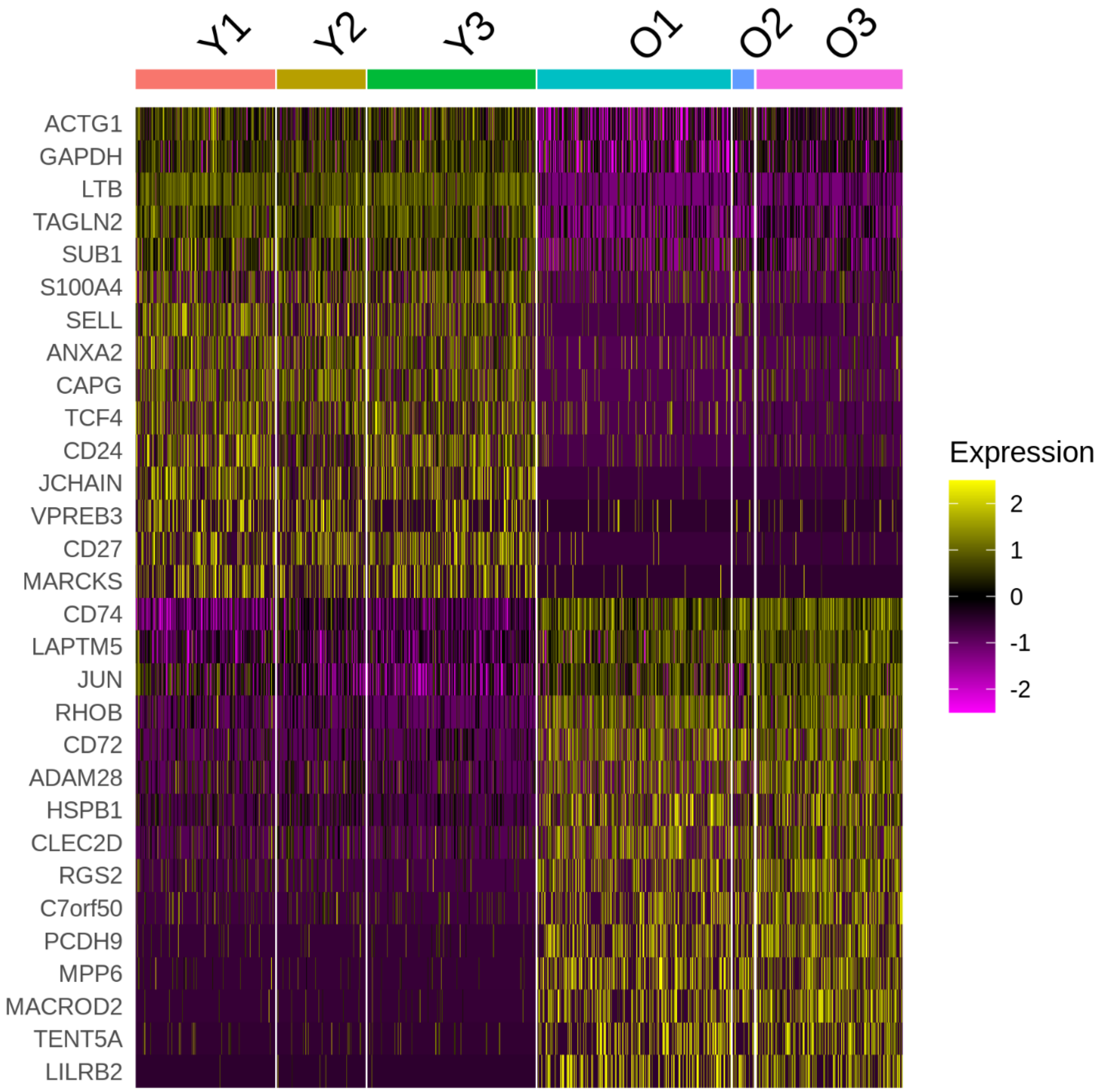

Supplementary Figure 4. Top 30 Differentially expressed genes between older and young adults enriched activated B cells at pre-vaccination baseline. Pseudobulk gene expression analysis was performed on the VFC clusters with the highest and lowest older-adult associated relative likelihood to find significantly differentially expressed genes with a Bonferroni adjusted p-value of 0.05.

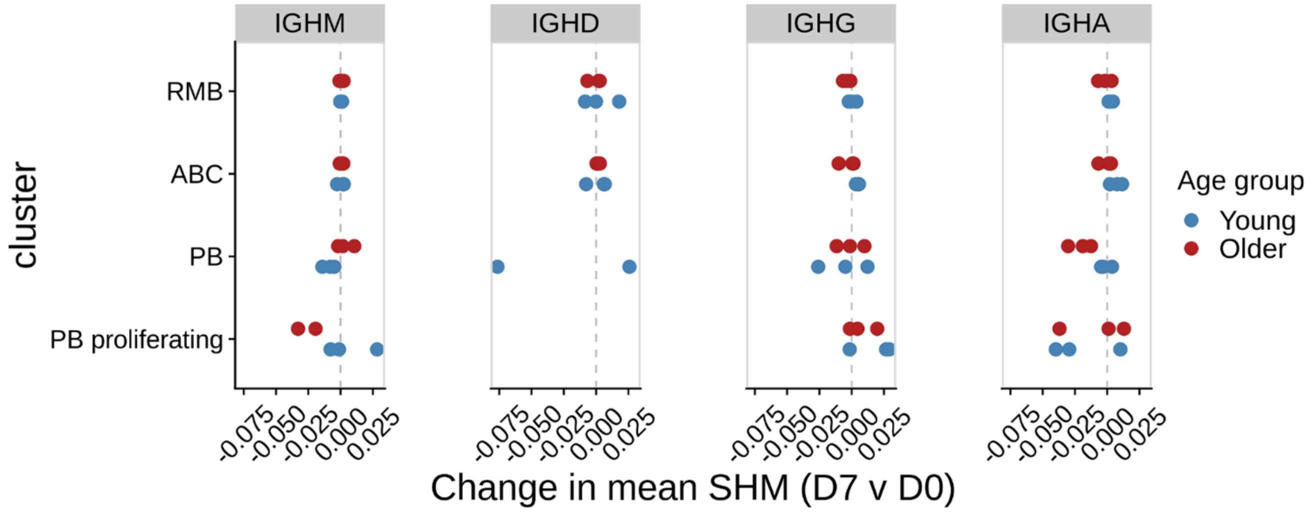

Supplementary Figure 5. Difference in the mean somatic hypermutation frequency between D7 and D0. Mutation frequency computed from the heavy chain V segments for each isotype and B cell types. The color indicates age group.

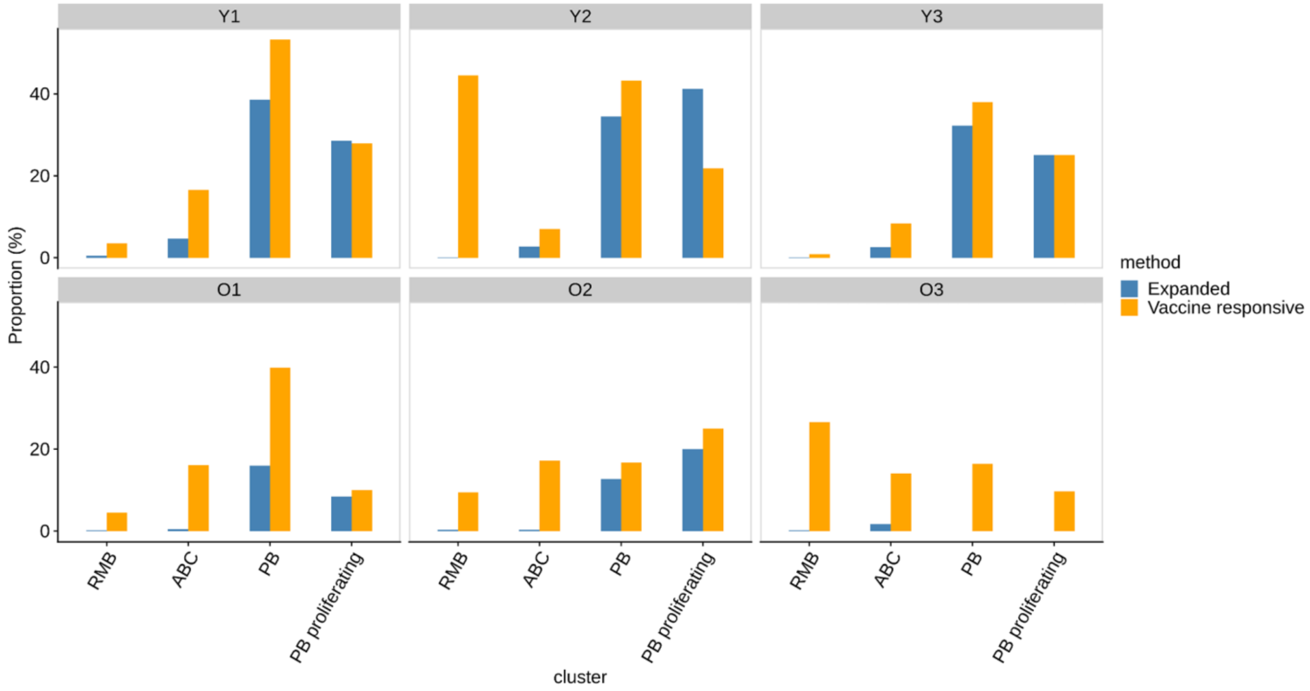

Supplementary Figure 6. Percentage of cell types identified as clonally expanded or vaccine responsive at D7.

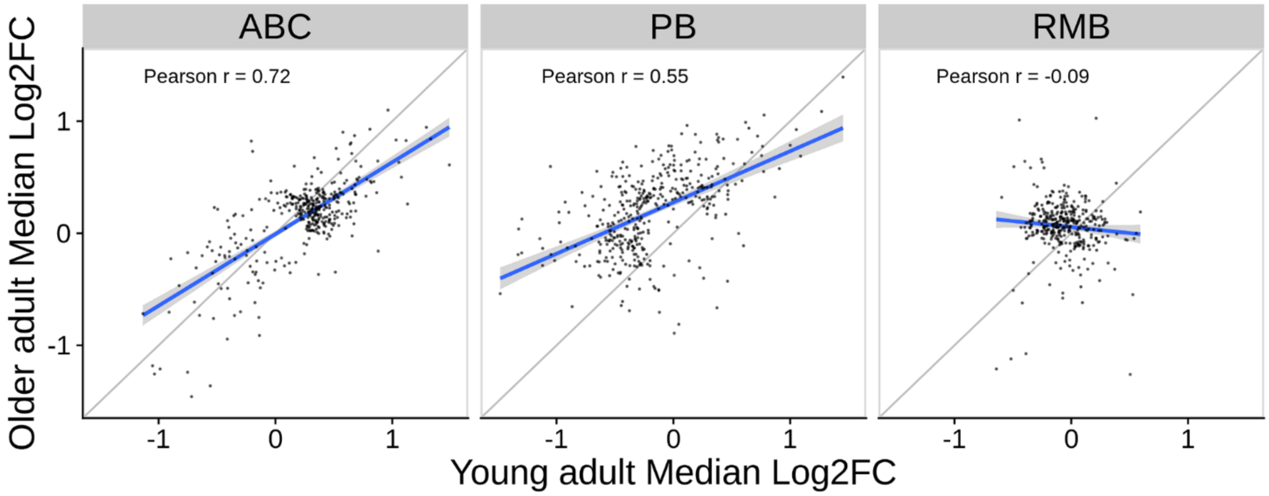

Supplementary Figure 7. Correlation of medians of log2 fold changes of differentially expressed genes between young and older subjects.

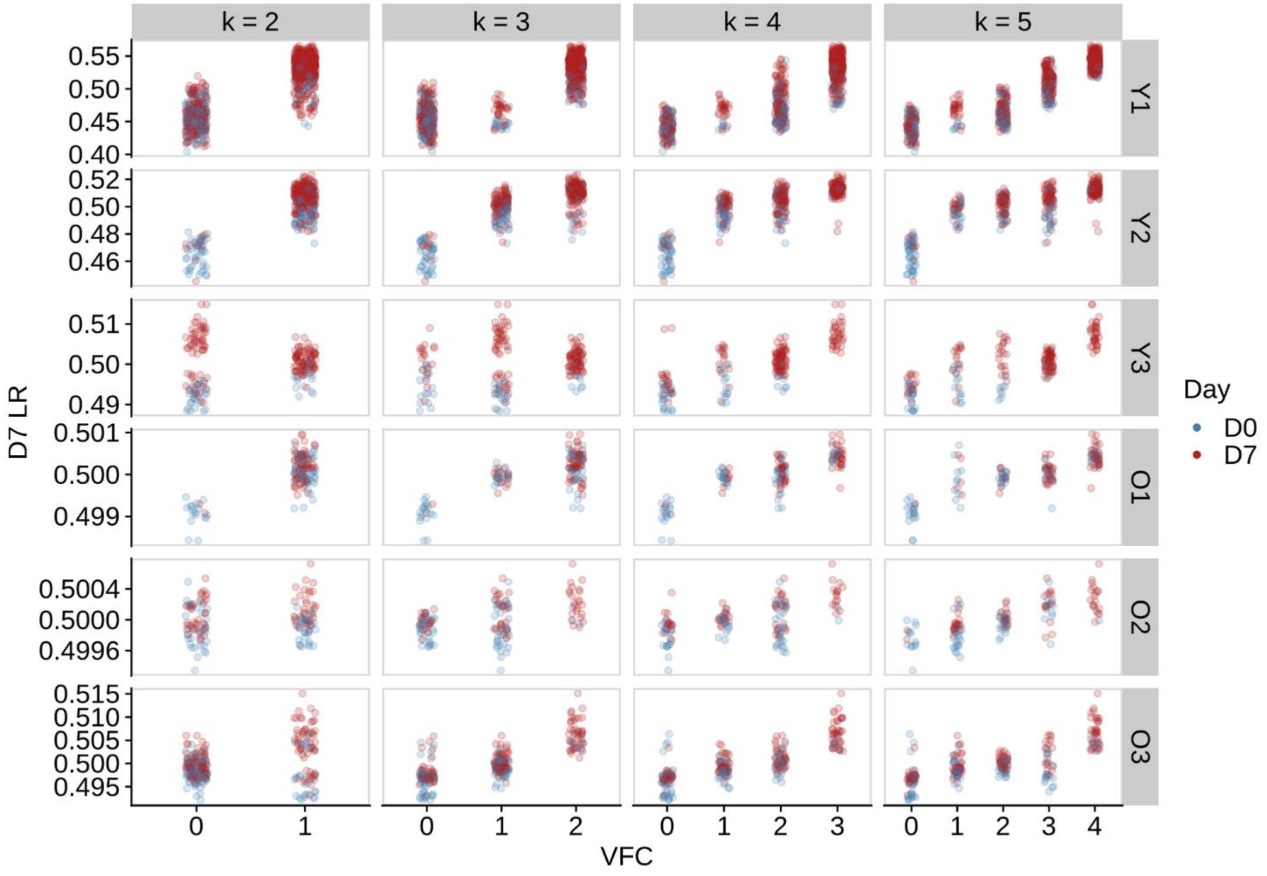

Supplementary Figure 8. Choice of the number of VFC clusters *k* to identify vaccine-responsive PB subpopulations. The x-axis is the cluster id and the y-axis is the relative likelihood of observing the cell in D7 relative to D0. The color indicates the time points. *k = 3* is the final choice of the number of clusters.

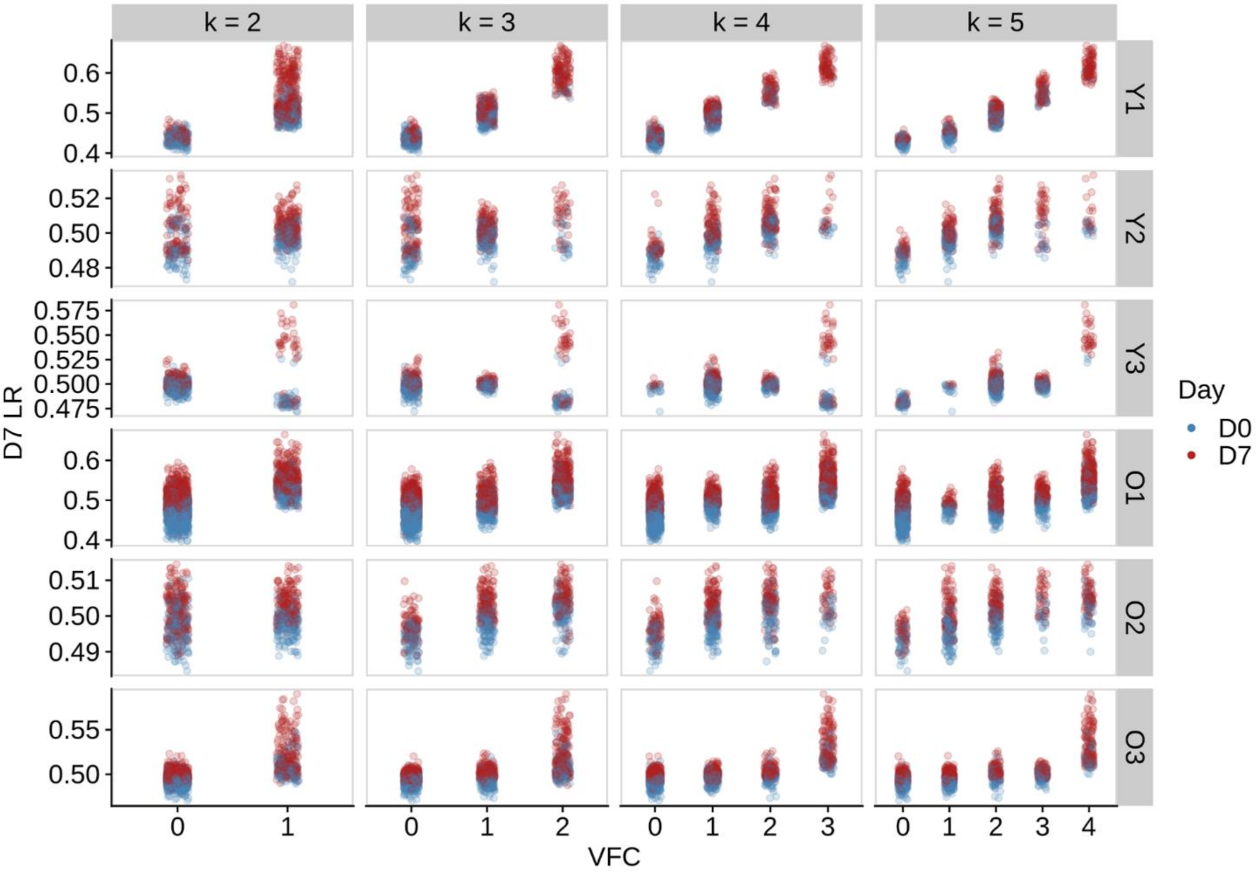

Supplementary Figure 9. Choice of the number of VFC clusters *k* to identify vaccine-responsive ABC subpopulations. The x-axis is the cluster id and the y-axis is the relative likelihood of observing the cell in D7 relative to D0. The color indicates the time points. *k = 3* is the final choice of the number of clusters.

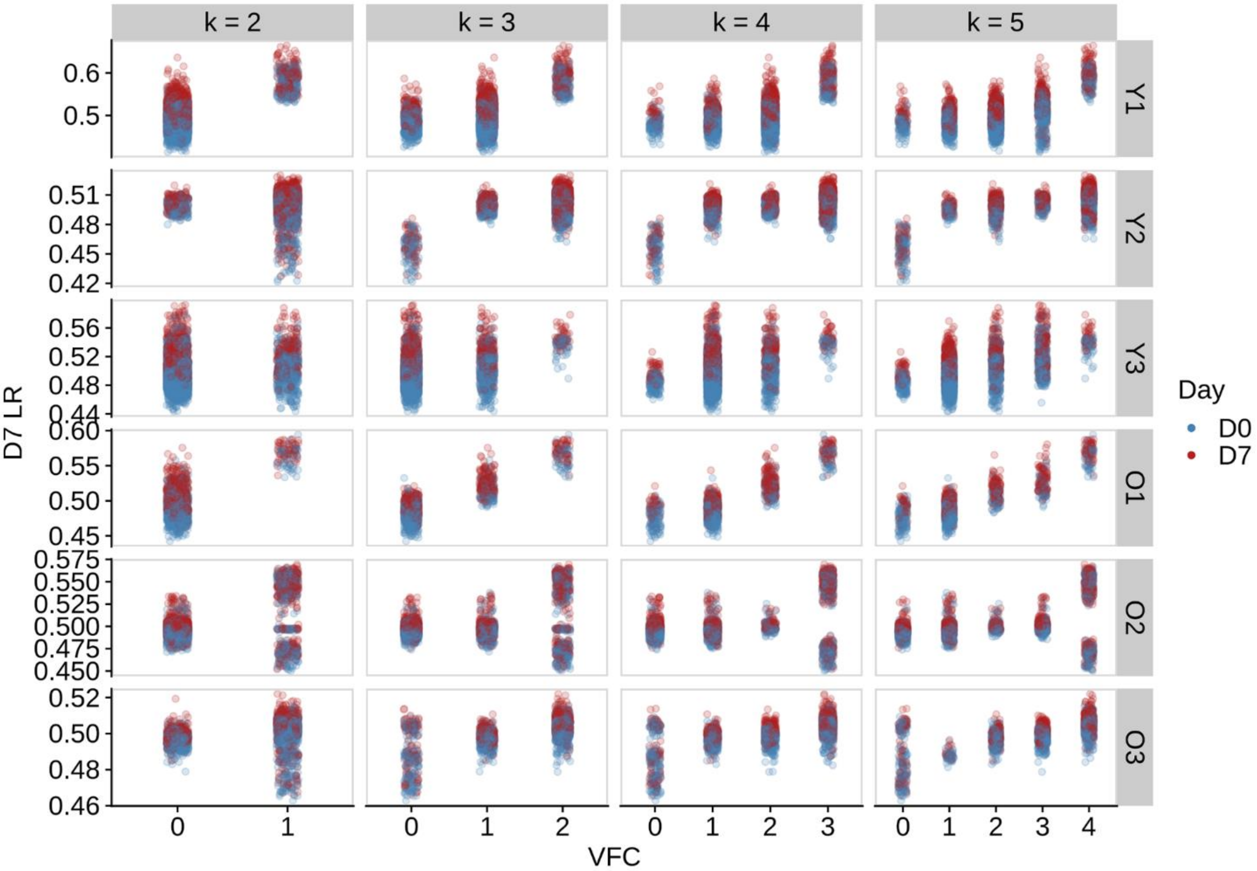

Supplementary Figure 10. Choice of the number of VFC clusters *k* to identify vaccine-responsive RMB subpopulations. The x-axis is the cluster id and the y-axis is the relative likelihood of observing the cell in D7 relative to D0. The color indicates the time points. *k = 3* is the final choice of the number of clusters.

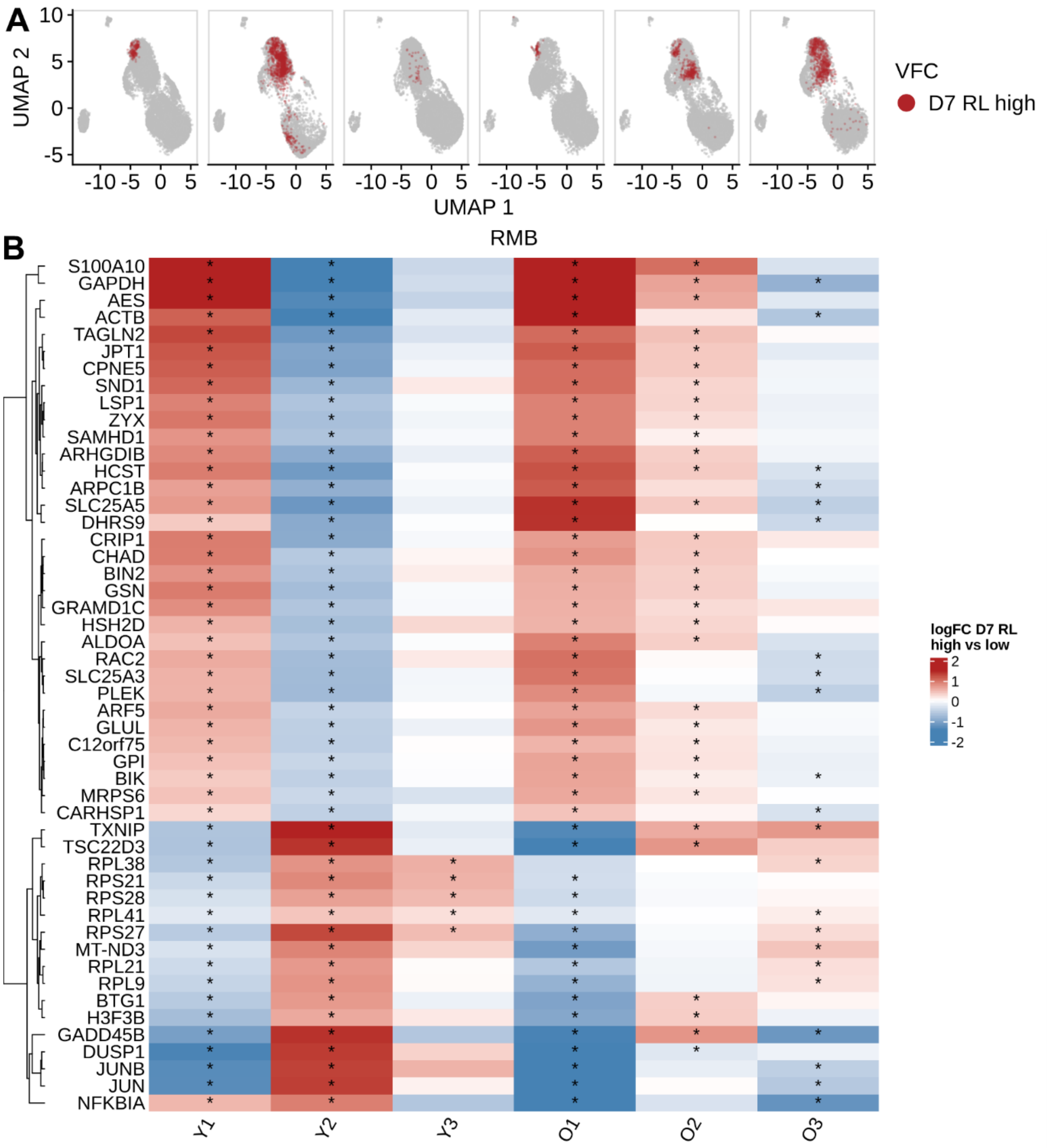

Supplementary Figure 11. Identifying vaccine-responsive subpopulations within resting memory B cells. (**A**) MELD was used to visualize the subset of resting memory B cells that increases most on day 7. (**B**) 34 differentially expressed genes between vaccine-responsive RMB and the rest of RMB. Wilcoxon rank-sum test was used to select differentially expressed genes comparing day 7 and day 0 samples of individual subjects. log-2 fold change of count values was computed for the differentially expressed genes. Genes that significantly differ between time points, and have an average log-2 fold change greater than 0.3 in at least one patient, were selected for visualization. The asterisk indicates an FDR-adjusted p-value for the Wilcoxon rank-sum test smaller than 0.05.

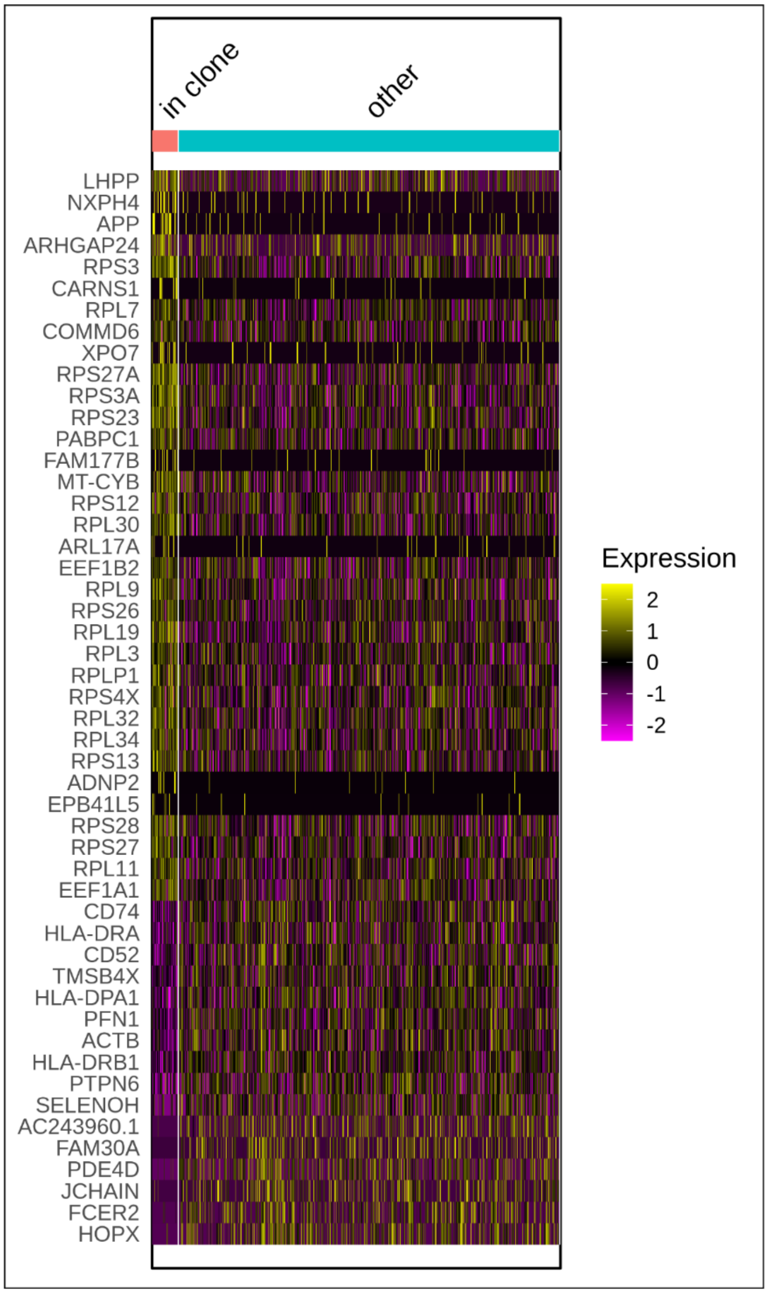

Supplementary Figure 12. Differentially expressed genes between the large, persistent clone and the resting memory IgG B cells in O3 at D0.
