# Supplementary material for: High-throughput single-cell profiling of B cell responses following inactivated influenza vaccination in young and older adults
Source: Aging (Albany NY). 2023 Jun 26;15(18):9250–74. doi: 10.18632/aging.204778 (PMC10564424; doi:10.18632/aging.204778)
Supplement: Supplementary Tables [file aging-15-204778-s002.pdf]

SUPPLEMENTARY TABLES

Supplementary Table 1. Sequencing depth information for scBCR-seq data for each sample.

ID	Day	Estimated number of cells	Number of read pairs	Mean read pairs per cell	Reads mapped to any V(D)J gene
O2	0	9020	29757249	3299	97.3%
O2	7	8297	22770915	2744	97.5%
Y2	0	11298	34464656	3050	97.8%
Y2	7	9646	28198097	2923	98.1%
O3	0	5817	30122076	5178	97.9%
O3	7	6032	27700863	4592	98.3%
O1	0	12222	27940821	2286	96.9%
O1	7	8513	30935126	3633	97.3%
Y1	0	16242	26511530	1632	97.2%
Y1	7	9543	26261853	2751	98.6%
Y3	0	16702	29206395	1748	96.2%
Y3	7	8431	35799555	4246	98.2%

Supplementary Table 2. Sequencing depth information for scRNA-seq data for each sample.

ID	Day	Estimated number of cells	Mean reads per cell	Median genes per cell	Number of reads	Reads mapped to genome
O2	0	11834	15779	1090	186740117	93.5%
O2	7	8510	23368	1120	198779306	93.2%
Y2	0	7695	36353	1162	279737332	93.6%
Y2	7	9222	28482	1211	262666599	93.3%
O3	0	6153	36582	1348	225090789	93.7%
O3	7	6649	34294	1330	228025482	93.1%
O1	0	15795	11728	927	185256017	93.1%
O1	7	9016	32639	1302	294281660	93.7%
Y1	0	13164	16033	1042	211069203	92.8%
Y1	7	11364	23571	1252	267869480	92.7%
Y3	0	16345	14726	1005	240707925	93.9%
Y3	7	7946	27764	1255	220615050	93.1%

Supplementary Table 3. Frequency of the cells identified as vaccine responsive from vertex frequency clustering analysis.

Subject	% RMB	% ABC	% PB
Y1	3.53	16.58	53.27
Y2	44.56	7.04	43.19
Y3	0.87	8.38	38.02
O1	4.45	16.08	39.86
O2	9.47	17.23	16.67
O3	26.52	14.05	16.41
